# P2X7 Receptor–Mediated Inflammation in Cardiovascular Disease

**DOI:** 10.3389/fphar.2021.654425

**Published:** 2021-04-29

**Authors:** Junteng Zhou, Zhichao Zhou, Xiaojing Liu, Hai-Yan Yin, Yong Tang, Xin Cao

**Affiliations:** ^1^ Department of Cardiology, West China Hospital, Sichuan University, Chengdu, China; ^2^ Division of Cardiology, Department of Medicine, Karolinska University Hospital, Karolinska Institutet, Stockholm, Sweden; ^3^ Laboratory of Cardiovascular Diseases, Regenerative Medicine Research Center, West China Hospital, Sichuan University, Chengdu, China; ^4^ School of Acupuncture and Tuina and International Collaborative Centre on Big Science Plan for Purinergic Signalling, Chengdu University of Traditional Chinese Medicine, Chengdu, China; ^5^ Acupuncture and Chronobiology Key Laboratory of Sichuan Province, Chengdu, China

**Keywords:** P2X7 receptor, inflammation, atherosclerosis, arterial hypertension, myocardial infarction

## Abstract

Purinergic P2X7 receptor, a nonselective cation channel, is highly expressed in immune cells as well as cardiac smooth muscle cells and endothelial cells. Its activation exhibits to mediate nucleotide-binding domain (NOD)-like receptor protein 3 (NLRP3) inflammasome activation, resulting in the release of interleukin-1 beta (IL-1β) and interleukin-18 (IL-18), and pyroptosis, thus triggering inflammatory response. These pathological mechanisms lead to the deterioration of various cardiovascular diseases, including atherosclerosis, arrhythmia, myocardial infarction, pulmonary vascular remodeling, and cardiac fibrosis. All these worsening cardiac phenotypes are proven to be attenuated after the P2X7 receptor inhibition in experimental studies. The present review aimed to summarize key aspects of P2X7 receptor–mediated inflammation and pyroptosis in cardiovascular diseases. The main focus is on the evidence addressing the involvement of the P2X7 receptor in the inflammatory responses to the occurrence and development of cardiovascular disease and therapeutic interventions.

## Introduction

Cardiovascular disease is a major cause of morbidity and mortality worldwide. Chronic inflammation is an important player in the pathogenesis of cardiovascular disease (Golia et al., 2014), which simultaneously impairs cardiac function, and continuously aggravates the symptoms of patients (Westermann et al., 2011).

Recent studies demonstrated that purinergic P2X7 receptors played an important role in a variety of cardiovascular diseases, including atherosclerosis (Piscopiello et al., 2013; Wernly and Zhou, 2020), arrhythmia after myocardial infarction (Gao et al., 2017), vascular remodeling (Mahdi et al., 2018; Hansen et al., 2020), and cardiac fibrosis (Zhou J. et al., 2020). P2X7 receptors are highly expressed in immune cells, including dendritic cells, mast cells, macrophages, and monocytes. As an ATP-gated ion channel, activation of the P2X7 receptor causes the release of interleukin-1 beta (IL-1β) and interleukin-18 (IL-18) recruitment by NLRP3, resulting in inflammatory response (Bracey et al., 2013; Stachon et al., 2017). A recent review made a summary on the role of P2X7 receptor–mediated endothelial dysfunction in hypertension, atherosclerosis, renal dysfunction, and cardiac and cerebral ischemia by promoting inflammatory responses (Shokoples et al., 2020). Apart from endothelial dysfunction, pyroptosis in cardiomyocytes was also demonstrated to be associated with the P2X7 receptor in heart injury (Liu et al., 2016). In our review, we will focus on key aspects of P2X7 receptor–mediated inflammation and pyroptosis in cardiovascular diseases, including atherosclerosis, arrhythmia, myocardial infarction, pulmonary vascular remodeling, and cardiac fibrosis, and the therapeutic value of targeting the P2X7 receptor.

## Relationship Among Adenosine Triphosphate, P2X7 Receptor, and Inflammation

ATP is a selective endogenous ligand and exerts as a natural agonist for P2X7 receptors. Under physiological conditions, the extracellular ATP concentration is about 30 nM in circulation. In pathological conditions, when the tissue is subjected to hypoxia or inflammation, a large amount of ATP is released, accumulated at the injured site or entered into circulation to activate P2X7 receptors and subsequent signaling (Bours et al., 2006).

A large and negatively charged ATP molecule is unable to directly diffuse across the lipid bilayer of the plasma membrane but can pass through the cell membrane *via* other regulatory or nonregulatory channels, such as connexins and pannexins (Novitskaya et al., 2016). During myocardial injury, ATP released from ischemic cardiomyocytes can bind to P2X7 receptors and activate platelets and inflammatory cells (Erlinge and Burnstock, 2008; Nishida et al., 2008; Burnstock and Pelleg, 2015). Activation of P2X7 receptors by ATP opens cation channels that are permeable to several cations, such as K^+^, Na^+^, and Ca^2+^, triggering a series of inflammatory responses (Baroja-Mazo et al., 2013; Sluyter, 2017). Moreover, continuous activation of P2X7 receptors forms nonselective membrane pores that allow molecules up to 900 kDa to pass, leading to cell membrane perforation and cell apoptosis (Hechler and Gachet, 2015). In addition, extracellular ATP opens K^+^ channels through ATP-gated P2X7 receptors, accelerating K^+^ outflow and thereby triggering NLRP3 inflammasome activation (He et al., 2017). The inflammasome is a multi-protein complex involved in the assembly and formation of cytoplasm by the pattern recognition receptor, mainly composed of receptor proteins (NLR or ALR family), apoptosis-related speck-like protein (ASC, apoptosis-associated speck-like protein containing CARD), and procaspase-1 (Atianand et al., 2013). It regulates the maturation and secretion of IL-1β and IL-18, as well as pyroptosis, playing an important role in the development of chronic inflammatory conditions, including cardiovascular disease (Dinarello, 2009; He et al., 2013; Mangan et al., 2018). The pro-inflammatory cytokines IL-1β and IL-18 are secreted by many cell types, and their gene expressions are regulated at both transcriptional and posttranslational levels. IL-1β precursors (pro–IL-1β) are inactive *in vivo*, and serine protease or caspase-1 processes them to the bioactive form (Afonina et al., 2015). Pattern recognition receptor (first signal, Signal 1), such as Toll-like receptors (TLRs) or cytokines receptors, leads to synthesis of the cytokine precursor pro–IL-1β and pro–IL-18 *via* NF-κB (Bauernfeind et al., 2009), whereas inflammasome (second signal, Signal 2) converts procaspase-1 into an enzyme-active form of caspase-1 (Franchi et al., 2009). Finally, caspase-1 processes pro–IL-1β and pro–IL-18 into their active forms, that is, IL-1β and IL-18, respectively, thus triggering inflammation (Kelley et al., 2019). Another study has shown that activation of P2X7 receptors resulted in a large influx of calcium ions, thus activating calmodulin-dependent protein kinase (CaMK) II and Ca^2+^-dependent phospholipase A2, and inducing the release of IL-1β (Xu and Liang, 2013).

Pyroptosis is a type of programmed cell death characterized by cellular swelling, rupture of membrane, release of cellular contents, and remarkable inflammatory response (Shi et al., 2015). Pyroptosis can be divided into caspase-1–dependent and caspase-independent pathways as follows: 1) under the stimulation of pathogens and bacteria, intracellular NLR recognizes these signals and activates caspase-1 by connecting ASC to pro–caspase-1. Gasdermin-D (GSDM-D), a pore-forming protein, cleaved by caspase-1, induces pyroptosis (Kayagaki et al., 2015; Shi et al., 2017). 2) Caspase-4/5/11 binds to LPS through the CARD domain inside the cell and triggers pyroptosis (Zhaolin et al., 2019). When P2X7 receptors activate the NLRP3 inflammasome in response to ATP, a circular platform is formed for the aggregation of ASC and caspase-1. Caspase-1 and other inflammatory caspases (caspase-4/5/11) cut GSDM-D into two fragments, resulting in the destruction of cell membranes through their pore-forming activity and promoting pyroptosis, as well as the releasing of IL-1β (Liu et al., 2016). IL-1β prolongs myocardial action potential duration (APD), decreases potassium current, and increases calcium sparks, oxidation, and phosphorylation of CaMK II, which facilitate the susceptibility of spontaneous systolic events and arrhythmias in cardiomyocytes (Monnerat et al., 2016).

**FIGURE 1:**
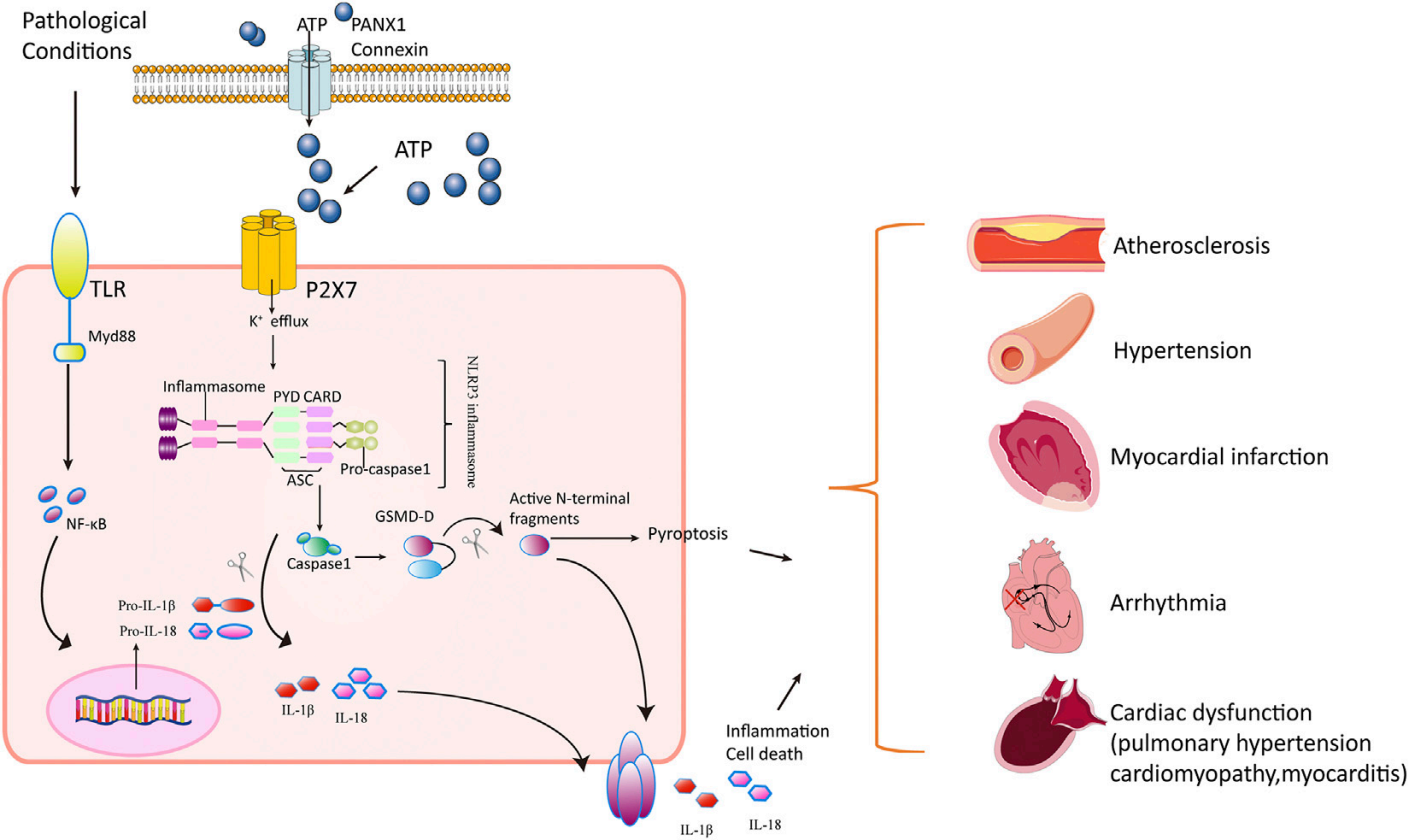
Mechanisms of P2X_7_ receptor’s action in cardiovascular disorders. In pathological stress including hypoxia/ischemia/hyperglycemia, the Toll-like receptors (TLRs) are activated, producing progenitors of inflammatory cytokines such as pro-IL-1β and pro-IL-18. Meanwhile, the P2X7 receptor is activated in response to ATP released through PANX1 and connexins in cardiomyocytes. The openness of P2X7 receptor leads to K^+^ efflux and Ca^2+^ influx, triggering NLRP3 inflammasome assembly (a circular platform consisting of NLR, ASC, and pro–caspase-1). NLRP3 inflammasomes convert pro–caspase-1 into active caspase-1. Caspase-1 cleaves the inactive pro–IL-1β and pro–IL-18 cytokines into active cytokines IL-1β and IL-18, respectively, and cuts GSDM-D into active N-terminal fragment. The active form of GSDM-D induces cell membrane disruption through their pore-forming activity and by promoting pyroptosis and releasing IL-1β and IL-18. The inflammatory response mediated by cytokines and pyroptosis contributes to pathology in atherosclerosis, hypertension, pulmonary hypertension, myocardial infarction, arrhythmia, cardiomyopathy, and autoimmune myocarditis.

Collectively, P2X7 receptors activated NLRP3 inflammasome in response to extracellular ATP, resulting in the release of IL-1β and IL-18, and play an important role in regulating inflammation and pyroptosis. This inflammatory response contributed to the pathology of cardiovascular diseases (Figure 1).

## Role of the P2X7 Receptor in Cardiovascular Diseases

### The P2X7 Receptor and Atherosclerosis

Atherosclerosis, characterized by lipid deposition and inflammatory response, is the major cause of coronary heart disease, cerebral infarction, and peripheral vascular disease. Once the lesions of atherosclerosis block the arterial lumen, tissues or organs supplied by the artery will show manifestations of ischemia or necrosis (Libby, 2013). One of the key features of atherosclerosis was the formation of atherosclerosis plaque that usually involves endothelial dysfunction followed by a series of complex pathological processes, including platelet activation and inflammatory cell aggregation. However, the specific mechanism underlying the formation of atherosclerosis plaque was not fully understood (Yang et al., 2017). Of importance, activation of P2X7 receptors has been shown to play a significant role in that process. Thus, expression of P2X7 receptors markedly increased in human carotid artery plaques and was correlated to the degree of coronary artery stenosis (Piscopiello et al., 2013; Shi et al., 2020). Gidlof et al.suggest that a common loss-of-function missense variant of the *P2RX7* gene may be associated with reduced ischemic heart disease in smokers (Gidlof et al., 2012). Therefore, it is reasonable to specify its involvement in plaque formation and rupture in atherosclerotic regions.

The P2X7 receptor is widely expressed in smooth muscle cells (SMCs) and endothelial cells, and the activation of the receptor in those cells plays a major role in the initiation and development of atherosclerosis (North, 2002; Franco et al., 2015). In atherosclerosis, turbulent blood flows at the lesion cause a significant increase in ATP to activate P2X7 receptors and their downstream signaling, such as p38 activation (Milner et al., 1990; Green et al., 2018). Impaired vascular regulation, oxidative stress, and inflammation can cause endothelial dysfunction in hyperglycemia and diabetes, which are risk factors for atherosclerosis (Zhou R. et al., 2020). Under the stimulation by high glucose and palmitic acid, the release of ATP activated endothelial P2X7 receptors, contributing to the production of pro-inflammatory factors and ROS, increase in cell permeability and cell adhesion molecules, and reduction of bioavailability of nitric oxide (NO) (Sathanoori et al., 2015; Lombardi et al., 2017; Stachon et al., 2017). In addition to endothelial cells and smooth muscle cells, increased expression of P2X7 receptors was also found in macrophages in the plaque of coronary atherosclerosis, suggesting the immune activity of P2X7 receptors. Knockout of the P2X7 receptor can reduce the infiltration of macrophages, caspase-1 activity, and the adhesion molecules, leading to small atherosclerotic degeneration (Lombardi et al., 2017; Stachon et al., 2017). Besides, lipid oxidation can activate NLRP3, and thus releases IL-1β and IL-18, accelerating the progression of atherosclerotic lesions, and reducing lipid oxidation can delay this progression (Leonarduzzi et al., 2012; Escalante et al., 2017). Studies have confirmed that activation of P2X7 receptors was involved in lipid metabolism of epithelial cells and immune cells, resulting in lipid oxidation and phagocytic clearance dysfunction (Costa-Junior et al., 2011; Guha et al., 2013). The P2X7 receptor knockout mice exhibited a lower cholesterol level and smaller atherosclerotic lesions than wide-type mice, suggesting the role of the P2X7 receptor in inflammation that is mediated by lipid oxidation (Duewell et al., 2010; Peng et al., 2015; Stachon et al., 2017). Interestingly, in Canakinumab Anti-Inflammatory Thrombosis Outcome Study (CANTOS), although IL-1 antagonist was associated with a reduction in cardiovascular events, cholesterol levels hardly altered, raising new challenges that whether the P2X7 receptor antagonists can reduce cholesterol levels (Ridker et al., 2017). It is also debatable whether atorvastatin played an anti-inflammatory role and reduced atherosclerosis by activating P2X7 receptors in endothelial cells, which is contradictory to the results of inflammation caused by endothelial P2X7 receptor activation (Mistafa et al., 2008; Lombardi et al., 2017). Therefore, the relationship between the P2X7 receptor and atherosclerosis warrants further study in the future.

### The P2X7 Receptor and Hypertension

Although the causes of hypertension are not fully understood, growing evidence has indicated that inflammation is not only associated with high blood pressure but also with the progression of this disease in recent years (Guzik et al., 2007; Kirabo et al., 2014). Studies have shown that activation of P2X7 receptors by uridine adenosine tetraphosphate led to vascular contraction (Zhou et al., 2015). Moreover, a single-nucleotide polymorphism (SNP) for the *P2RX7* gene was associated with blood pressure, especially for rs591874, which was related to nocturnal diastolic blood pressure (Palomino-Doza et al., 2008). Among Chinese postmenopausal women, single-nucleotide polymorphism (rs3751143-C) of the *P2RX7* gene was linked to a reduced risk of essential hypertension (Gong et al., 2019). Of note, plasma ATP levels in hypertensive mice began to increase from the third day after the induction of hypertension, which was consistent with the higher plasma ATP levels in hypertensive patients than in normal subjects (Zhao et al., 2019). Also, sustained shear stress can induce the release of ATP in endothelial cells, which may lead to the activation of P2X7 receptors (Yamamoto et al., 2000). These results indicate that the P2X7 receptor is involved in the occurrence and development of hypertension.

Hypertensive rats on a high-salt diet exhibited increased expression of P2X7 receptors, whereas the P2X7 receptor knockout mice showed decreased systolic and diastolic blood pressure (Ji et al., 2012). Likewise, IL-1β in cultured macrophages significantly increased after treatment with the P2X7 receptor agonist BzATP in salt-sensitive rats when compared with salt-resistant rats (Ji et al., 2012). In addition to blood pressure, the P2X7 receptor was also closely related to hypertension-related renal injury. Chronic activation of the renin–angiotensin system promoted the occurrence of hypertension, renal microvascular dysfunction, hypoxia, and inflammation. In this process, activation of P2X7 receptors can intensify microcirculatory obstruction, regional ischemia, and hypoxia, accelerating the progression of renal injury induced by angiotensin II (Menzies et al., 2015). Blocking P2X7 receptors may benefit renal function, which can reduce renal vascular resistance, mainly in the anterior glomerular artery and arteriole. The possible mechanisms might be as follows: first, the antagonistic effect of P2X7 receptors increased myelin perfusion, oxygenation, and attenuated renal inflammation; second, the P2X7 receptor blockade enhanced the pressure–urinary sodium excretion reaction (Arulkumaran et al., 2011; Solini et al., 2013); last, in an angiotensin II–dependent hypertension rat model, the P2X7 receptor antagonist A438079 reduced efferent and afferent arteriole resistance, suggesting the role of the P2X7 receptor in glomerular hemodynamics (Franco et al., 2017).

It is worth noting that hypertension was prevalent in patients with autoimmune diseases, in which both antigen-presenting cells (APCs) and T cells caused higher blood pressure (Madhur et al., 2010; Wilck et al., 2017). The elevated concentration of extracellular ATP acted as a “danger signal” to mobilize immune cells, especially through the P2X7 receptor that upregulated CD86 expression in APCs. Of note, hydrolyzing ATP or blocking the P2X7 receptor can effectively block the upregulation of CD86 and decrease the reactivity of T cells under hypertension (Zhao et al., 2019). Interestingly, IL-1β antagonist significantly reduced blood pressure in one kidney/deoxycorticosterone acetate (DOCA)/salt-induced hypertension in rats, despite the fact that monoclonal antibody against IL-1β did not reduce blood pressure or incident hypertension during follow-up in CANTOS (Ling et al., 2017; Ridker et al., 2017; Rothman et al., 2020). Therefore, the P2X7 receptor, as the upstream of IL-1β, may become a promising target for the treatment of hypertension in the future.

### The P2X7 Receptor and Pulmonary Arterial Hypertension

PAH is a disease with poor prognosis and high mortality, which is accompanied by a progressive increase of pulmonary artery resistance and eventually right heart failure (Simonneau et al., 2019). This disease is characterized by the abnormal structure of pulmonary artery endothelial cells (PAECs), secretion of inflammatory factors, proliferation of pulmonary artery smooth muscle cells (PASMCs), thickening of the pulmonary artery wall, and narrowing of its lumen, leading to the remodeling of pulmonary artery structure (Vonk et al., 2019). Currently, the five-year mortality rate is as high as 59% for the lack of specific treatment for PAH (Boucly et al., 2017). Therefore, it is urgent to explore the pathogenesis and new therapeutic targets for PAH.

Inflammation is involved in the development and progression of PAH. Pulmonary arterial lesions are infiltrated by macrophages, T cells, B cells, and other inflammatory cells in the damaged artery of PAH patients (Price et al., 2012). Several other studies found that the levels of pro-inflammatory cytokines, such as C-reactive protein (CRP), IL-1β, IL-6, IL-8, IL-10, monocyte chemotactic protein 1 (MCP-1), tumor necrosis factor α, and interferon, significantly increased in the plasma of patients with idiopathic PAH (Rabinovitch et al., 2014; Pezzuto et al., 2015). Despite the heterogeneity among species, the P2X7 receptor is expressed in isolated PAECs and intact pulmonary vascular systems from the lungs of mice and humans (Cai et al., 2020). In animal models of pulmonary hypertension induced by monocrotaline (MCT), the activation of P2X7 receptors is accompanied by an increase in NLRP3 inflammasome and IL-1β. At the same time, activated P2X7 receptors colocate with myeloid cells and vascular cells (Yin et al., 2017b; Duan et al., 2018). Additionally, studies have confirmed the role of ATP–P2X7 receptor axis in the development of pulmonary inflammation, and the proliferation and migration of vascular smooth muscle cells (Robinson et al., 2006; Lucattelli et al., 2011). The above evidence implies that P2X7 receptors’ activation may be associated with PAH by increasing a strong inflammation response.

Blockade of the P2X7 receptor ameliorated PAH to some extent. In a rat model of PAH induced by MCT, administration of P2X7 receptor antagonist A74003 for 2 weeks reversed pulmonary vascular remodeling and significantly reduced NLRP3 inflammasome, TNF-α, and IL-1β levels (Yin et al., 2017b). In another study using the same model, Brilliant Blue G (BBG), a P2X7 receptor antagonist, not only alleviated the remodeling of pulmonary vessels and function but also reduced the levels of pro-inflammatory cytokine IL-1β and tumor necrosis factor α through the P38/MAPK signaling pathway (Duan et al., 2018). Interestingly, a novel inhibitor of P2X7 receptor PKT100 improved the right ventricle (RV) contractility and systolic function and survival in the PAH mice model induced by bleomycin, with no effect on pulmonary arterial pressure and pulmonary vascular remodeling (Hansen et al., 2020). This provides a new perspective for current treatment strategies that target both the RV function and pulmonary vessels, which may improve the prognosis of PAH patients. To sum up, the inhibition of the P2X7 receptor exerted anti-inflammatory and anti-remodeling effects on PAH, suggesting that the P2X7 receptor may be a potential therapeutic target. Although studies on the role of P2X7 receptor in PAHs are not completely understood, further exploration on novel disease mechanisms and validation of this receptor in clinical trials are requested.

### The P2X7 Receptor and Myocardial Infarction

The main cause of acute myocardial infarction (AMI) is coronary thrombosis and continuous blood flow obstruction, secondary to rupture of unstable atherosclerotic plaque. Large amounts of ATP and oxidative stress products (reactive oxygen species) are released from cardiomyocytes and endothelial cells, leading to P2X7-mediated NLRP3 inflammasome formation and activation around the border area of infarcted tissues (Cheung et al., 2015; Hesse et al., 2017). It should be noted that activation of NLRP3 inflammasome in cardiomyocytes induces caspase-dependent pyroptosis rather than producing IL-1β (Toldo et al., 2018; Zeng et al., 2019; Wang et al., 2020). This is supported by the fact that fibroblasts and endothelial cells produce IL-1β in ischemia/reperfusion injury, but not cardiomyocytes (Heid et al., 2013; Sandanger et al., 2013; Liu et al., 2014). Additionally, epicardial-derived cells (EPDCs) also play an important role in the P2X7–NLRP3–IL-1β axis. After MI in the adult heart, EPDCs are activated, proliferated, and migrated into the damaged myocardial layer. They differentiate into fibroblasts, vascular smooth muscle cells, pericytes, and adipocytes, and secrete many signaling molecules that affect myocardial regeneration (Cai et al., 2008; Dube et al., 2017; Quijada et al., 2019). CD73 on EPDCs degrades extracellular ATP and nicotinamide adenine dinucleotide (NAD) to adenosine, thereby activating the A2B receptor. In turn, in addition to IL-6 formation, activation of the A2B receptor also enables intracellular vesicles to release ATP and NAD, forming a positive feedback. A large amount of ATP/NAD activates P2X7 receptors, leading to the formation of the NLRP3 inflammasome and release of IL-1β (Hesse et al., 2017). Also, the NAD from EPDCs can bind to the enzyme ARTC2 expressed on the regulatory T (Treg) cell, activating P2X7 receptors by transferring ADP-ribose to cell surface. The downstream signaling pathway, L-selectin, and CD27 will be cleaved, causing phosphatidylserine to flip outward and leading to Treg cell apoptosis (Seman et al., 2003; Aswad et al., 2005; Adriouch et al., 2008; Scheuplein et al., 2009). Treg can protect cardiomyocytes from apoptosis, reduce inflammation and adverse ventricular remodeling after infarction, and promote cardiac healing (Matsumoto et al., 2011; Meng et al., 2016).

It is also worth noting that SNPs for the *P2RX7* gene played a role in hypertension but not in myocardial infraction. None of the SNPs (A1513C, rs208294, and rs3751143) for the *P2RX7* gene was associated with mortality in patients with heart failure, a common consequence of myocardial infarction (Eslick et al., 2009; Pasqualetti et al., 2017).

Accordingly, inhibition of P2X7 receptors may reduce infarcted areas and improve cardiac function and survival by suppressing inflammation. After MI, treatment of small interfering RNA (siRNA) or P2X7 receptors inhibitor can prevent the formation of NLRP3 inflammasome and limit the infarcted area (Barth et al., 2010). Moreover, Mezzaroma et al. demonstrated that inhibition of cryopyrin or P2X7 receptors could blunt caspase-1 activation and assembly of inflammasomes, subsequently reducing cell death and reversing ventricular remodeling (Mezzaroma et al., 2011). Canakinumab, a monoclonal antibody targeting IL-1β, has been shown to reduce the adverse cardiac event (myocardial infarction and/or stroke) by 15% in patients with a prior MI (Ridker et al., 2017). Therefore, despite the limited clinical data, targeting P2X7 receptor to reduce inflammation may provide a new direction for future studies.

### The P2X7 Receptor and Arrhythmia

After ischemia-reperfusion injury or MI, arrhythmia is one of the most common complications, putting patients at risk for deterioration of cardiac function and sudden cardiac death (Arevalo et al., 2016). The release of various inflammatory cytokines induced a continuous inflammatory response in cardiac tissue, and thus generated pro-arrhythmic substrates of tissue fibrosis and electrical remodeling (Eisenhut and Wallace, 2011). NLRP3 and IL-1β played an important role throughout the whole process. After cardiac injury, IL-1β was elevated by TLR and NLRP3 inflammasomes, and it prolonged APD, decreased outward potassium current, and increased calcium spark, oxidation, and phosphorylation of CaMK II (Cerrone et al., 2012; Anumonwo and Pandit, 2015). These changes resulted in impaired contractility and the propensity of arrhythmia for cardiomyocytes. In addition, inflammatory responses were also involved in sympathetic nerve regeneration by increasing sympathetic nerve density and tone. Sympathetic hyperinnervation promoted cell apoptosis and deteriorated cardiac function (Lindholm et al., 1987; Hoffmann et al., 2013). The sympathetic nerve of the heart branches from stellate ganglia. After MI, the expression of P2X7 receptor increased in stellate ganglia, indicating that the P2X7 receptor was involved in neural remodeling (Kong et al., 2013; Tu et al., 2016). These observations suggest that the P2X7 receptor–NLRP3–IL-1β axis is associated with arrhythmia.

In animal studies, blockade of the P2X7 receptor or its downstream regulators has been proven to be effective for arrhythmias. The P2X7 receptor inhibitor A740003 or IL-1β antagonist Anakinra can blunt sympathetic hyperinnervation and sympathetic sprouting by suppressing the infiltration of macrophages and production of IL-1β and nerve growth factor (NGF) (Yin et al., 2017a). Inhibiting P2X7 receptors with short hairpin RNA (shRNA) improved sympathetic hyperinnervation and cardiac remodeling by inhibiting the Akt and ERK1/2 pathways and NF-κB activation, which in turn promoted IL-1β production (Gao et al., 2017). In addition, NONRATT021972 siRNA targeting the P2X7 receptor restored the abnormal distribution of sympathetic nerve fibers around the ischemic myocardium and sympathetic overexcitation, and increased norepinephrine and epinephrine concentrations and blood pressure (Tu et al., 2016). Besides, De Jesus et al. demonstrated that IL-1β antagonist Anakinra significantly improved cardiac conduction function and intracellular Ca^2+^ concentration, decreased transmembrane potential and Ca^2+^ alternating amplitude, and reduced the incidence of spontaneous ventricular arrhythmia. The mechanism underlying the myocardial infarction–induced arrhythmia may be related to increased expression levels of connexin 43 and Ca^2+^-ATPase in the sarcoplasmic reticulum (De Jesus et al., 2017). Together, therapeutic interventions targeting P2X7 receptor may protect against cardiac arrhythmia.

### The P2X7 Receptor and Cardiomyopathy

Cardiomyopathy is an anatomic and pathologic diagnosis associated with a high incidence of morbidity and mortality. Cardiomyopathy may be secondary (e.g., infiltrative, toxic, and inflammatory) (Wexler et al., 2009). The pathophysiological mechanism of cardiomyopathy is still unclear. However, cardiac fibrosis has been successively confirmed to be involved in its pathophysiological process, among which inflammatory reaction plays a key role (Nishida and Otsu, 2017). IL-1β and IL-18 lead to the initiation of pyroptosis and fibrosis in diabetic cardiomyopathy (Vandanmagsar et al., 2011; Luo et al., 2014). In a calcineurin-induced structural heart disease mouse model, elevated NLRP3 mRNA levels, cardiac hypertrophy, inflammation, and ventricular dilation were observed (Bracey et al., 2013). Meanwhile, in transverse aortic constriction (TAC)-induced dilated cardiomyopathy, the expression of P2X7 receptor, NLRP3 inflammasome, and its downstream effectors significantly increased, accompanied by impaired cardiac function and collagen deposition (Zhou J. et al., 2020). Likewise, SNP rs28360451-A for the *P2RX7* gene is involved in hypertrophic cardiomyopathy, and loss of function mutation will cause a defective phenotype (Biswas et al., 2019).

The P2X7 receptor–NLRP3–IL-1β axis is involved in the pathophysiological process of cardiac fibrosis. In pathological conditions, plenty of collagen I and III are produced by activated fibroblasts under the long-term stimulation of IL-18 and IL-1β, leading to the excessive extracellular matrix, cardiac fibrosis, and remodeling (Turner, 2016). Additionally, under TGF-β stimulation, NLRP3 inflammasomes increase in cardiac fibroblasts, which promote the activation of receptor-associated Smad (R-SMad) in myofibroblasts. Activated R-Smads (RSmad 2/3) bind to co-SMAD (Smad 4) to form a transcription complex to promote the expression of fibrogenic genes (Bracey et al., 2014). Moreover, caspase-1, the product of NLRP3, and other caspases (caspase-4/5/11) cut GSDMD into two fragments, in which the amino-terminal one damages cell membranes through its pore-forming activity, thereby promoting pyroptosis and the release of IL-1β (Liu et al., 2016).

Targeting P2X7 receptor directly alleviated structural and functional abnormalities in cardiomyopathy. H3 relaxin, a member of the insulin-like growth factor superfamily, can inhibit collagen synthesis of cardiac fibroblasts in the high-glucose environment by attenuating the activation of the ROS and P2X7 receptor–mediated NLRP3 inflammasome (Zhang et al., 2017; Zhang et al., 2018). In addition, in the TAC mouse model, treatment with the P2X7 receptor antagonist BBG reduced cardiac fibrosis and improved systolic function by inhibiting the expression of NLRP3 and IL-1β. Inhibition of downstream regulators of NLRP3 inflammasome also prevents the progression of cardiomyopathy. It has been proven that the knockout of NLRP3 or IL-1β receptor antagonists improved the mitochondrial structure of muscle fibers and ultimately restored cardiac function of cardiomyopathy *via* reducing the extracellular matrix (Bracey et al., 2013; Luo et al., 2014). Overall, the blockade of P2X7 receptor may ameliorate the progression of cardiomyopathy by suppressing inflammation and fibrosis, especially in cardiac fibroblasts.

### The P2X7 Receptor and Autoimmune Myocarditis

Autoimmune myocarditis is characterized by a series of inflammatory responses accompanied by the necrosis of cardiomyocytes and infiltration of monocytes. No specific treatment is available until now (Suzuki et al., 2007; Fung et al., 2016). Heart biopsy from patients with acute myocarditis showed that inflammasomes were only observed in the patient group when compared with control subjects who died without cardiac conditions and were higher in patients with New York Heart Association (NYHA) III-IV than in those with NYHA I-II (Toldo et al., 2014). In the primary mouse submandibular gland, P2X7 receptors activated NLRP3 inflammasome and released IL-1β, contributing to autoimmunity (Khalafalla et al., 2017). These data suggest that P2X7 receptor may be involved in autoimmune myocarditis through pro-inflammatory cascades.

In a murine experimental autoimmune myocarditis (EAM) model, P2X7 receptors were activated and the EAM model showed impairment of cardiac systolic function and infiltration of inflammatory cells 21 days after EAM induction. The application of P2X7 receptor antagonist A74003 restored the systolic function by suppressing the CD4^+^ T cells and macrophage infiltration, as well as the IL-1β mRNA expression (Zempo et al., 2015). It is worth mentioning that the P2X7 receptor knockout mice exhibited the phenotype of dilated cardiomyopathy despite lower IL-1β and IL-17 levels than its wide-type ones in an anti-M2 muscarinic receptor–induced autoimmune cardiomyopathy model (Martinez et al., 2015). Thus, further studies are needed to better understand the safety and efficacy of the P2X7 receptor in autoimmune myocarditis.

## Conclusions and Perspectives

Numerous studies suggest that activation of P2X7 receptors plays a crucial role in cardiovascular disease, and targeting P2X7 receptor is an effective tool to alleviate the progression of cardiovascular diseases such as atherosclerosis, hypertension, pulmonary hypertension, myocardial infarction, arrhythmia, cardiomyopathy, and autoimmune myocarditis. Further attention should be paid to potential roles for P2X7 receptor activation in cardiac allograft rejection (Wu et al., 2016; Vergani et al., 2013; D’Addio et al., 2018). The mechanisms mainly involve the P2X7 receptor–mediated inflammatory response induced by cytokines and pyroptosis. However, P2X7 inhibitors showed limited efficacy in clinical trials of other inflammatory diseases. In a clinical trial of rheumatoid arthritis, the P2X7 inhibitors AZD9056 (AstraZeneca) and CE-224535 (Pfizer) did not significantly improve symptoms (Keystone et al., 2012; Stock et al., 2012). Similarly, in Crohn’s disease, AZD9056 hardly lowers the inflammatory markers, although it improved the patients’ activity index scores (Eser et al., 2015). Given the good tolerance of P2X7 inhibitors in clinical trials and the favorable efficacy obtained in various animal models, P2X7 receptor inhibition could represent a novel approach to the treatment of cardiovascular disease in humans. However, clinical trials by targeting the P2X7 receptor to alleviate cardiovascular disease have not been conducted. Therefore, it is necessary to conduct in-depth research on the role of P2X7 receptor in cardiovascular disease and translate these findings from animal models into clinical studies.
